# N-Heterocyclic carbenes

**DOI:** 10.3762/bjoc.11.268

**Published:** 2015-12-07

**Authors:** Steven P Nolan

**Affiliations:** 1 Chemistry Department, College of Science, King Saud University, Riyadh 11451, Saudi Arabia

The N-heterocyclic carbenes (NHC) now hold a preferred-ligand status in organic and organometallic chemistry. Their role in catalysis continues to grow. When the editorial staff at the Beilstein Journal of Organic Chemistry contacted me to act as a guest editor for the thematic series “N-Heterocyclic carbenes”, I was more than happy to accept what appeared to me to be a simple and exciting endeavor. Indeed, the job was made simple as the number of research groups focusing on this topic continues to grow and today the literature is flooded with one use or another of these fascinating, stabilizing ligands.

To state here what a great influence these ligands have had on modern synthetic chemistry in providing unique catalytic tools (but also in view of their stabilizing effects due to their steric and electronic tunable properties) might be unnecessary. The use of these ligands now extends to numerous fields spanning from fine chemicals to polymer synthesis. The area of main group chemistry has also benefited from these ligands as stabilizing entities.

Since the seminal work of Bertrand [1] and Arduengo [2] (and mostly post-1994–1995) the field has underwent fantastic advances. Carbenes such as 1,3-dimesityl-1,3-dihydro-2*H*-imidazol-2-ylidene (IMes) and the 2,6-diisopropylphenyl analogue (IPr) have become commonplace, replacing tertiary phosphanes as modifying ligands on metals.

The field continues to experience tremendous development, with a considerable number of publications still reporting new reactions that are catalyzed and controlled by the carbenes as ligands or as organocatalysts. The action of state-of-the-art catalysts are nowadays better understood through detailed mechanistic work, which permits the design of ever-better performing catalytic systems. This design/mechanistic study/redesign cycle will ensure the continued evolution of the field. The area of NHC-based research is a worldwide effort, as exemplified by contributions in this NHC-focused Thematic Series.

This compendium again highlights the diversity of fields that are affected and enhanced by NHCs. It is my hope that such a collection of contributions will inspire readers in novel and exciting directions. I never tire of being amazed at the ingenuity of researchers in using these entities as building blocks for progress.

Steven P. Nolan

Riyadh, November 2015
